# Divide and Conquer: Stratifying Training Data by Tumor Grade Improves Deep Learning-Based Brain Tumor Segmentation

**DOI:** 10.3389/fnins.2019.01182

**Published:** 2019-11-05

**Authors:** Michael Rebsamen, Urspeter Knecht, Mauricio Reyes, Roland Wiest, Raphael Meier, Richard McKinley

**Affiliations:** ^1^Support Center for Advanced Neuroimaging (SCAN), University Institute of Diagnostic and Interventional Neuroradiology, Inselspital, Bern University Hospital, University of Bern, Bern, Switzerland; ^2^Graduate School for Cellular and Biomedical Sciences, University of Bern, Bern, Switzerland; ^3^Institute for Surgical Technology and Biomechanics, University of Bern, Bern, Switzerland; ^4^Healthcare Imaging A.I. Lab, Insel Data Science Center, Inselspital, Bern University Hospital, Bern, Switzerland

**Keywords:** magnetic resonance imaging, brain tumors, automatic segmentation, deep learning, training strategy, data stratification

## Abstract

It is a general assumption in deep learning that more training data leads to better performance, and that models will learn to generalize well across heterogeneous input data as long as that variety is represented in the training set. Segmentation of brain tumors is a well-investigated topic in medical image computing, owing primarily to the availability of a large publicly-available dataset arising from the long-running yearly Multimodal Brain Tumor Segmentation (BraTS) challenge. Research efforts and publications addressing this dataset focus predominantly on technical improvements of model architectures and less on properties of the underlying data. Using the dataset and the method ranked third in the BraTS 2018 challenge, we performed experiments to examine the impact of tumor type on segmentation performance. We propose to stratify the training dataset into high-grade glioma (HGG) and low-grade glioma (LGG) subjects and train two separate models. Although we observed only minor gains in overall mean dice scores by this stratification, examining case-wise rankings of individual subjects revealed statistically significant improvements. Compared to a baseline model trained on both HGG and LGG cases, two separately trained models led to better performance in 64.9% of cases (*p* < 0.0001) for the tumor core. An analysis of subjects which did not profit from stratified training revealed that cases were missegmented which had poor image quality, or which presented clinically particularly challenging cases (e.g., underrepresented subtypes such as IDH1-mutant tumors), underlining the importance of such latent variables in the context of tumor segmentation. In summary, we found that segmentation models trained on the BraTS 2018 dataset, stratified according to tumor type, lead to a significant increase in segmentation performance. Furthermore, we demonstrated that this gain in segmentation performance is evident in the case-wise ranking of individual subjects but not in summary statistics. We conclude that it may be useful to consider the segmentation of brain tumors of different types or grades as separate tasks, rather than developing one tool to segment them all. Consequently, making this information available for the test data should be considered, potentially leading to a more clinically relevant BraTS competition.

## 1. Introduction

Gliomas are primary brain tumors which arise from glial cells. According to the World Health Organization (WHO) classification of tumors of the central nervous system (CNS) (Louis et al., 2016), they can be grouped into different tumor grades based on the underlying histology and molecular characteristics. Increasing tumor grade indicates the increasing malignancy of the tumor. Glioma are managed depending on grade, with treatment strategies ranging from tumor resection followed by combined radio- and chemotherapy to a “watch and wait” approach (Stupp et al., 2005; Grier, 2006). Glioblastoma are the most aggressive type of glioma (WHO grade IV) and make up 45% of all gliomas (Ostrom et al., 2014). The prime imaging technique in brain tumor diagnostics is Magnetic Resonance Imaging (MRI) (Essig et al., 2012). Standard acquisition protocols used to perform initial diagnosis and treatment monitoring include T1-weighted, T1-weighted gadolinium-enhanced, T2-weighted, and T2-weighted with fluid attenuated inversion recovery (FLAIR) sequences (Wen et al., 2010; Ellingson et al., 2015). The typical radiological appearance of a glioblastoma features a disrupted blood-brain barrier causing ring-enhancing lesions with central necrosis and peritumoral edema. In contrast, low-grade astrocytic tumors exhibit typically no contrast enhancement and are missing central necrosis (Pierallini et al., 1997).

In the case of glioblastoma, recent studies led to the discovery of a profound genetic heterogeneity among, and even within, tumors (Verhaak et al., 2010; Sottoriva et al., 2013). It has been shown that the underlying genetic and molecular heterogeneity can be associated with variations in imaging phenotype such as changes in tumor compartment volumes (Lai et al., 2013; Grossmann et al., 2016), contrast enhancement (Carrillo et al., 2012; Treiber et al., 2018), radiomic signatures (Gevaert et al., 2014), and tumor location (Carrillo et al., 2012; Ellingson et al., 2012). The imaging appearance of glioblastoma can further be altered by treatment causing radiation necrosis (Mullins et al., 2005) and pseudoprogression and -response (Hygino da Cruz et al., 2011), respectively. As a consequence, a machine learning segmentation algorithm needs to be capable of generalizing across this heterogeneity of glioblastoma imaging phenotypes.

Brain tumor segmentation is a well-investigated topic with a vast amount of available methods and yearly organized MICCAI Brain Tumor Segmentation (BraTS) Challenges since the year 2012 (Menze et al., 2015; Bakas et al., 2017c), serving as a public platform for algorithm comparison. With the rise of deep learning, brain tumor segmentation methods experienced significant gains in performance (Bakas et al., 2018). One of the central promises of deep learning methods is that they can be fed with raw data and are capable of automatically uncovering the underlying representation relevant for the task at hand (e.g., segmentation) from that data (LeCun et al., 2015). As a consequence, the time-consuming and error-prone manual engineering of features traditionally used in machine learning has been rendered obsolete. Recently, it was shown for vision tasks that model performance increases logarithmically based on volume of training data (Sun et al., 2017). This aligns with the general notion that more training data leads to a better generalization of a machine learning algorithm. Within the context of BraTS Challenges, deep learning methods are usually trained *ad hoc* on all of the available data, disregarding underlying latent factors such as genetic characteristics or even tumor grades. Although the tumor type is available to the challenge participants for the training data, this information is withheld for the validation and test data. Since part of the BraTS dataset is coming from *The Cancer Imaging Archive* (TCIA) (Bakas et al., 2017a,b,c), additional relevant information such as e.g., patient's gender, mutation subtypes [Isocitrate dehydrogenase (IDH), 1p19q co-deletion] and methylation status of MGMT-promotor could potentially be added as well.

The metric of choice for algorithm comparison in biomedical image segmentation challenges is the Dice coefficient, which was used in 92% of the 383 segmentation tasks reported in Maier-Hein et al. (2018). Predominantly, the Dice coefficient is reported in terms of summary statistics (mean/median) over patient cases and model comparison is performed on the basis of such summary statistics (metric-based ranking). Recently, the BraTS Challenge adopted a case-based ranking scheme. While metric-based rankings lead to more robust rankings than case-based rankings (Maier-Hein et al., 2018), it can be argued that distinct performance differences for individual patients may be obfuscated.

We hypothesize that deep learning methods for brain tumor segmentation can be significantly improved by taking into account latent factors along with tumor image appearance during model training. The purpose of this study is to demonstrate the impact of including prior knowledge of a particular latent factor (tumor grade) on the performance of a recently published, top-ranked deep learning method (McKinley et al., 2019a). Furthermore, the impact is studied employing both a metric-based and case-based rank analysis.

The idea of leveraging prior information about tumor grades to improve segmentation has been presented as an extended abstract to the *International Conference on Medical Imaging with Deep Learning (MIDL)* along with preliminary results (Meier et al., 2019).

## 2. Materials and Methods

### 2.1. Study Data

The study is based on publicly-available data of the BraTS 2018 Challenge (Menze et al., 2015; Bakas et al., 2017c). In particular, the training dataset was used, which includes 75 patients with low-grade glioma (LGG) and 210 patients with high-grade glioma (HGG). The imaging data encompasses four MR image sequences (T1-weighted, T1-weighted with contrast agent, T2-weighted, and T2-weighted FLAIR sequences), which are part of the consensus recommendations for a standardized brain tumor imaging protocol in clinical trials (Ellingson et al., 2015). The imaging data stem from 19 different institutions, which relied on different MR scanners and acquisition protocols. Manual segmentations of three tumor compartments were available: contrast-enhancing tumor, non-enhancing/necrosis combined, and edema. The regions which were considered for evaluation in the BraTS 2018 challenge as well as in the study at hand were: contrast-enhancing tumor, tumor core (all compartments except edema), and whole tumor (all compartments). More details on the preprocessing and the evolution of the BraTS dataset can be found in Bakas et al. (2017c).

### 2.2. Automatic Segmentation

The network architecture used for the automatic segmentation is equivalent to the model ranked third in the BraTS 2018 challenge (McKinley et al., 2019a). In brief, it is a U-net-style structure with densely connected blocks of dilated convolutions. The segmentation is performed slice-wise where the input data includes the two neighboring slices from below and above from all four image modalities (i.e., input dimension is *batch* × 4 × 5 × 192 × 192). The final segmentation is the result of ensembling the predictions from all three directions (sagittal, axial, and coronal).

In a pre-processing step, the data are first normalized to zero mean and unit variance. Data augmentation consists of a combination of randomly flipping the images along the midline and random rotations [*angle*~*U*(−15, +15)] around all principal axis. Additionally, the standardized voxel intensities are randomly shifted [*amount*~*N*(0, 0.5)] and scaled [*factor*~*N*(1, 0.2)].

The networks were trained with a *focal loss* function, *RMSprop* as optimizer with a cosine-annealing learning rate schedule, and a batch-size of two.

### 2.3. Stratified Model Training

Three different models were trained independently, each with a five-fold cross-validation: A baseline model with all available training data (number of samples *N* = 285), an HGG-only model (*N* = 210), and an LGG-only model (*N* = 75). Network architecture and hyperparameters were the same for all models which were trained on a *Nvidia GeForce GTX 1080 Ti* GPU with 11GB memory over 80 epochs. Qualitatively, the performance on the validation-set was saturating with no observable overfitting (see Figure S1).

### 2.4. Statistical Analysis

The statistical analysis was performed using *R* with the *stats* package version 3.5.1 (R Core Team, 2018). For comparison of spatial overlap of estimated tumor segmentations with manual ground truth data, the Dice coefficient was used. Segmentation performance in terms of Dice coefficient of the different deep learning models was summarized by descriptive statistics (median, interquartile range). Case-based rank analysis included computation of percentage of improved patient cases for given pairing of deep learning models. The stratified models were compared to the baseline by means of paired difference tests: differences between the cross-validated classifiers were examined on HGG cases only, on LGG cases only, and on the whole dataset (using the combined results of the stratified LGG and HGG classifiers). Non-parametric tests were employed due to the rank-based form of the data. The significance level of the analysis was set to α=0.05.

## 3. Results

### 3.1. Quantitative Analysis

**Figure 1:**
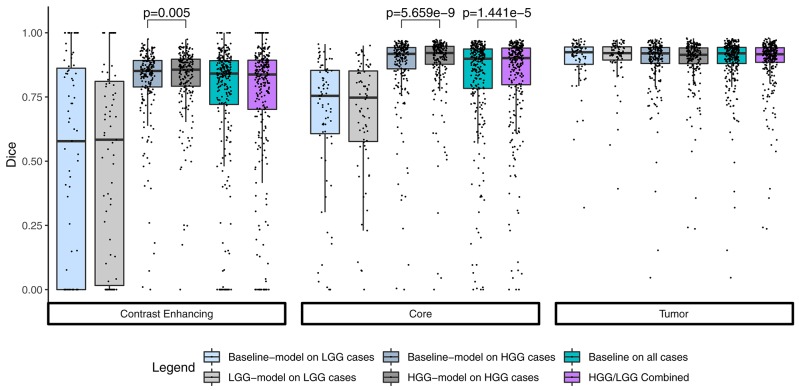
Summary statistics for the segmentation of the three compartments by means of a Tukey boxplot. *p*-values indicate statistically significant (*p* < 0.05) improvements determined by a one-sided Wilcoxon signed rank test.

Summary statistics for the segmentation performance in terms of Dice coefficient are shown in Figure 1. The baseline model reached a median Dice of 0.841 (1.5 × IQR = 0.465–1.000) for the contrast enhancing compartment, 0.899 (0.554–1.000) for the core, and 0.920 (0.786–1.000) for the whole tumor. Comparable, the combined results from the separately trained HGG/LGG models were 0.838 (0.415–1.000) for contrast enhancing, 0.902 (0.584–1.000) for core, and 0.916 (0.800–1.000) for tumor.

**Figure 2:**
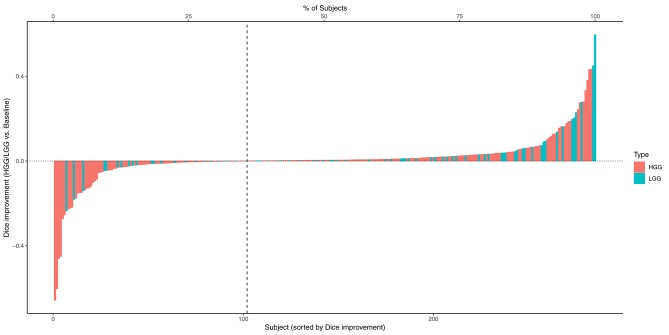
Absolute change of Dice coefficients of the tumor core for each subject. Positive changes were observed for the subjects on the right side of the dashed vertical line.

The combined results of the two separately trained models showed an improvement for the segmentation of the tumor core in 64.9% (*p* < 0.0001) of the subjects compared to the baseline model (Table 1). No statistically significant changes were observed for the other compartments. This performance gain originates primarily from the HGG cases where 70.3% of the subjects showed an improved segmentation for the tumor core and 58.5% of the subjects also for the contrast enhancing compartment. From the 183 subjects that showed an improved segmentation of the core, 26 increased by a Dice of 0.1 or more. Conversely, from the 99 subjects with a declined performance, 21 decreased by a Dice of −0.1 or more (Figure 2).

**Table 1:** Ratio in % of better performing subjects compared to baseline.

	**CE**		**Core**		**Tumor**	
	**% Subjects**	***p***	**% Subjects**	***p***	**% Subjects**	***p***
LGG vs. Baseline	41.7	0.877	49.3	0.454	54.7	0.208
HGG vs. Baseline	**58.4**	**0.005**	**70.3**	**5.659e-09**	46.7	0.877
HGG/LGG vs. Baseline	54.6	0.127	**64.9**	**1.441e-05**	48.8	0.725

*Statistical significance is determined by a one-sided Wilcoxon signed rank test. Bold numbers indicate statistically significant (p < 0.05) results. CE: contrast enhancing*.

### 3.2. Qualitative Analysis of Selected Cases

From Table 1 it is evident that, especially for high-grade glioma, stratified training leads to improved segmentation performance. In order to further investigate this aspect, a visual review of selected cases was performed. To identify cases mostly affected by the stratified training, Dice coefficients between the segmentations of the two models (baseline vs. HGG) were calculated. Cases with a Dice agreement <0.8 of the tumor core between the baseline and stratified models were selected for a qualitative manual inspection followed by a review with a board-certified neuroradiologist with more than 8 years of experience in brain tumor diagnostics. In order to render the visual review more systematic, we define three categories of causes for variability in tumor segmentation performance: 1. The input data generated by the imaging process, which is affected by the idiosyncrasies of the MR scanner, potential image artifacts and patient motion, and image preprocessing. 2. The manual ground truth segmentation. 3. The tumor phenotype (e.g., IDH-mutant tumor, presence of intratumoral hemorrhage, or cystic components) which causes distinctively different image appearances.

**Figure 3:**
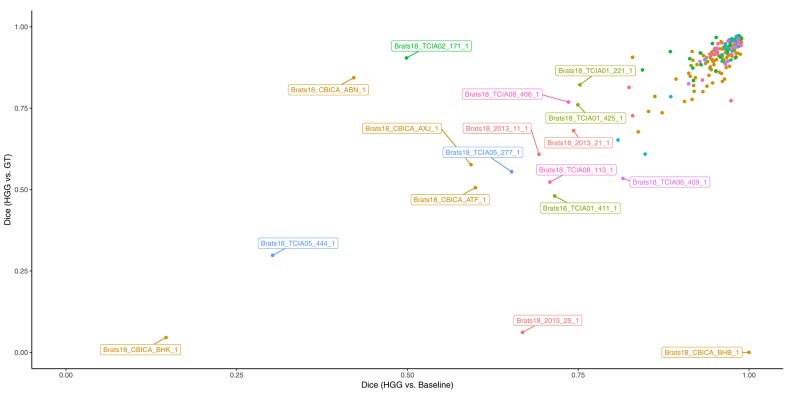
Performance of the HGG-only model for the tumor core (y-axis) and agreement with the baseline model (x-axis). Subjects with a label were visually reviewed. Colors indicate the center (2013, CBICA, TCIA01-08).

In Figure 3 the obtained Dice coefficients between the segmentation results of the HGG model for the tumor core and the ground truth were plotted against the Dice coefficients between the results of the HGG model and the segmentation of the baseline model, which was trained on all available data. We can broadly define four different territories in the scatterplot: The upper right corner which contains cases for which both models achieved high segmentation performance. If we move to the upper left corner, we encounter cases for which the HGG model achieved high segmentation performance with discrepancies when compared to the results of the baseline model. If we move from the upper right corner to the lower right corner, we encounter cases for which the HGG model agreed with the segmentation result of the baseline model but did not agree with the ground truth result. Finally, the lower left corner contains cases for which the segmentation results of the HGG model did neither agree with the ground truth nor with the segmentation of the baseline model. The corresponding scatterplots for the other two compartments can be found in Figures S2, S3. The identified outlier cases are listed in Table 2 with the segmentation performance of the two models and an assessment category. Below we present the observations based on a visual review for a selection of the identified outliers. Visualizations for the remaining outliers can be found in Figures S4–S14.

**Table 2:** Performance of selected cases for the two models.

**Subject**	**Assessment**	**Dice Baseline-model**			**Dice HGG-model**		
		**CE**	**Core**	**Tumor**	**CE**	**Core**	**Tumor**
Brats18_2013_11_1	1	0.14	0.45	0.90	0.17	0.61	0.89
Brats18_2013_21_1	3	0.80	0.83	0.94	0.76	0.68	0.94
Brats18_2013_25_1	3	0.18	0.10	0.90	0.25	0.06	0.90
Brats18_CBICA_ABN_1	2	0.84	0.41	0.82	0.79	0.84	0.83
Brats18_CBICA_ATF_1	3	0.69	0.73	0.69	0.65	0.51	0.63
Brats18_CBICA_AXJ_1	2	0.79	0.35	0.90	0.79	0.58	0.90
Brats18_CBICA_BHB_1	2	0.00	0.00	0.15	0.00	0.00	0.24
Brats18_CBICA_BHK_1	2	0.01	0.00	0.05	0.25	0.05	0.24
Brats18_TCIA01_221_1	2	0.76	0.88	0.95	0.48	0.82	0.95
Brats18_TCIA01_411_1	1	0.07	0.24	0.71	0.23	0.48	0.64
Brats18_TCIA01_425_1	–	0.26	0.58	0.75	0.68	0.76	0.78
Brats18_TCIA02_171_1	2	0.89	0.47	0.95	0.89	0.90	0.95
Brats18_TCIA04_343_1	2	0.69	0.73	0.74	0.59	0.61	0.66
Brats18_TCIA05_277_1	3	0.42	0.37	0.85	0.56	0.55	0.90
Brats18_TCIA05_444_1	3	0.39	0.96	0.94	0.54	0.30	0.89
Brats18_TCIA06_409_1	–	0.52	0.53	0.89	0.50	0.53	0.86
Brats18_TCIA08_113_1	1	0.91	0.36	0.97	0.79	0.52	0.92
Brats18_TCIA08_406_1	1	0.65	0.63	0.88	0.68	0.77	0.90

*Assessment after a qualitative review with a neuroradiologist. Assessment 1: Issue with input image quality, 2: Possible problem with ground truth, 3: Special phenotype, GT: ground truth, CE: contrast enhancing*.

**Figure 4:**
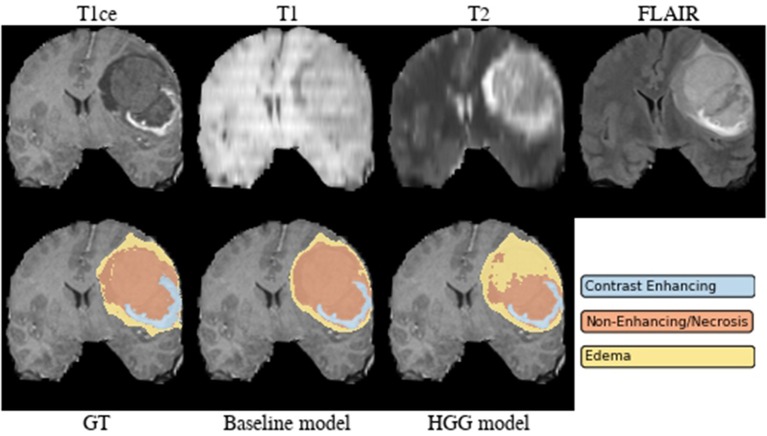
Brats18_2013_21_1.

**Brats18_2013_21_1** (Figure 4). The baseline model provided superior performance for segmenting the tumor core in this HGG example. The lesion exhibits a large non-enhancing tumor mass (typically seen in LGG) and we speculate that the presence of LGG cases in the baseline model led to the improved tumor core segmentation performance when compared to the HGG model's result. The appearance of the tumor is further complicated by the presence of cystic components, which exhibit a homogeneous signal that is strongly hypointense in T1-weighted and hyperintense in T2-weighted images.

**Figure 5:**
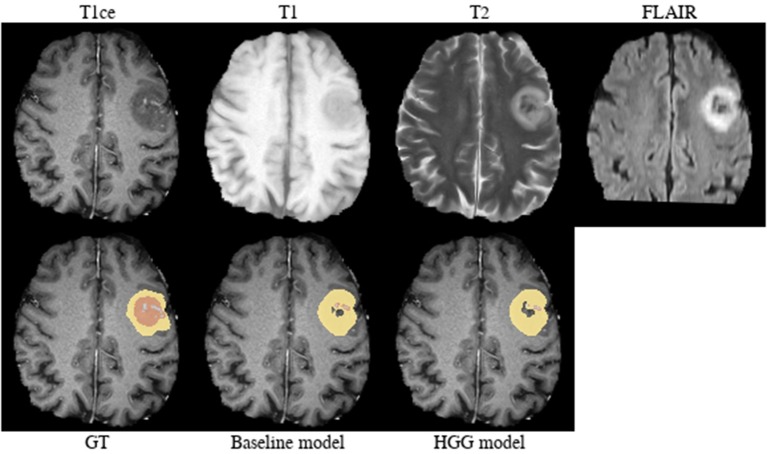
Brats18_2013_25_1.

**Brats18_2013_25_1** (Figure 5). Both models failed to segment the tumor core for this HGG case. The tumor core contains strongly hypointense areas in the T2-weighted and FLAIR images with corresponding heterogeneous signal intensity in the T1-weighted image. When considering the T1/T1c-weighted images, one can observe the presence of recruited blood vessels. This image appearance may indicate the presence of an intratumoral hemorrhage.

**Figure 6:**
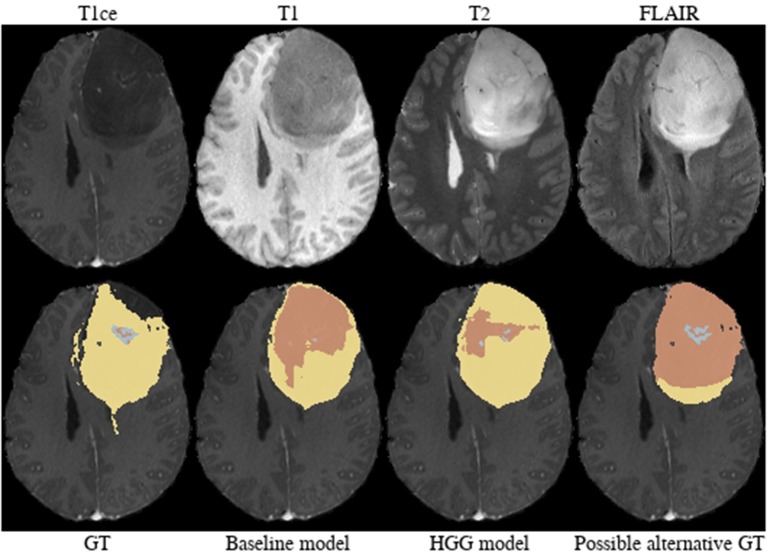
Brats18_CBICA_AXJ_1.

**Brats18_CBICA_AXJ_1** (Figure 6). The segmentation of the core from the HGG model is closer to ground truth. The tumor was indicated to be an HGG. However, the provided ground truth segmentation seems to be missing part of the tumor mass in the frontal lobe. Furthermore, we argue that a large part of the lesion corresponds to non-enhancing tumor rather than edema. We base this assumption on the heterogeneous appearance in the T2-weighted images and more importantly the strong cortical space-occupying effect together with a distortion of the gray/white matter junction. In contrast, edema would preserve the gray/white matter junction as well as the cortical ribbon and propagate along the white matter fiber tracts. A possible alternative for ground truth is shown in Figure 6.

**Figure 7:**
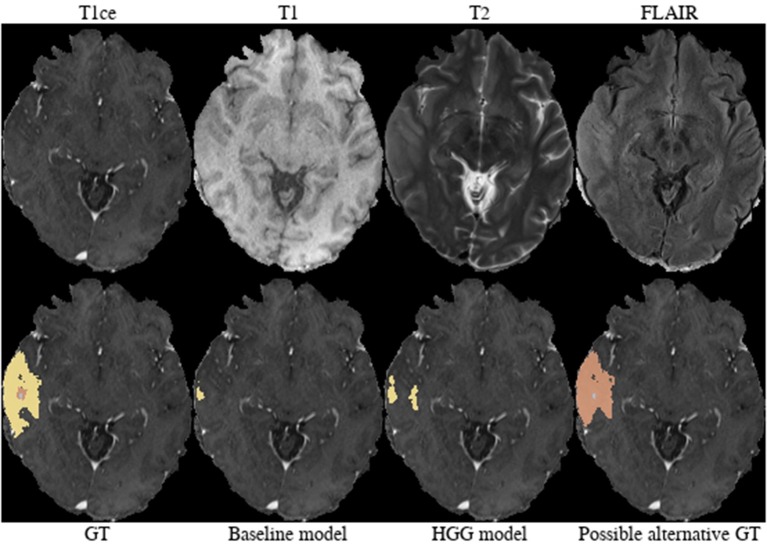
Brats18_CBICA_BHB_1.

**Brats18_CBICA_BHB_1** (Figure 7). Both models failed completely to segment the lesion for this HGG case. However, the provided ground truth segmentation seems to overestimate the presence of edema. While we agree on the whole tumor segmentation, we argue that the present T2-weighted hyperintensity indicates the presence of non-enhancing tumor rather than edema. Similarly to case Brats18_CBICA_AXJ_1 the gray/white matter junction is distorted. This is especially evident when considering the unaffected contralateral hemisphere. The poor segmentation performance of both models might be the result of an under-representation of training samples with such a subtle tumor core which is potentially ambiguously labeled in other cases as well.

**Figure 8:**
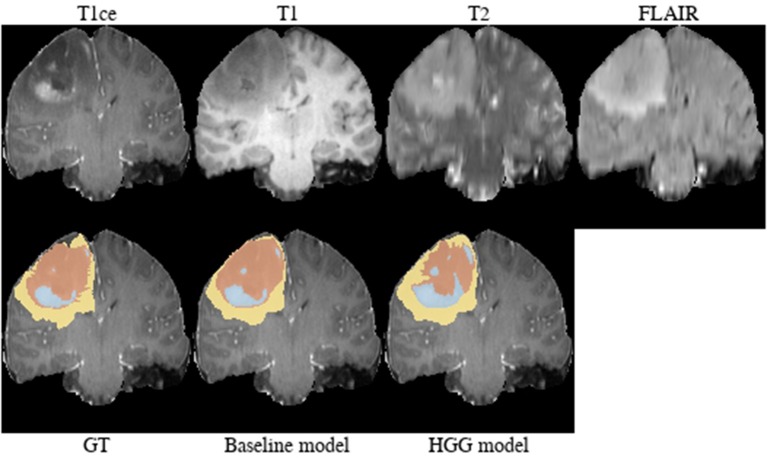
Brats18_TCIA01_221_1.

**Brats18_TCIA01_221_1** (Figure 8). The baseline model provided the better tumor core segmentation for this HGG case. However, when comparing the segmentation of the contrast-enhancing tumor of the HGG model, we argue that the ground truth segmentation slightly undersegments it. This is clearly visible for the enhancing rim next to the midline.

**Figure 9:**
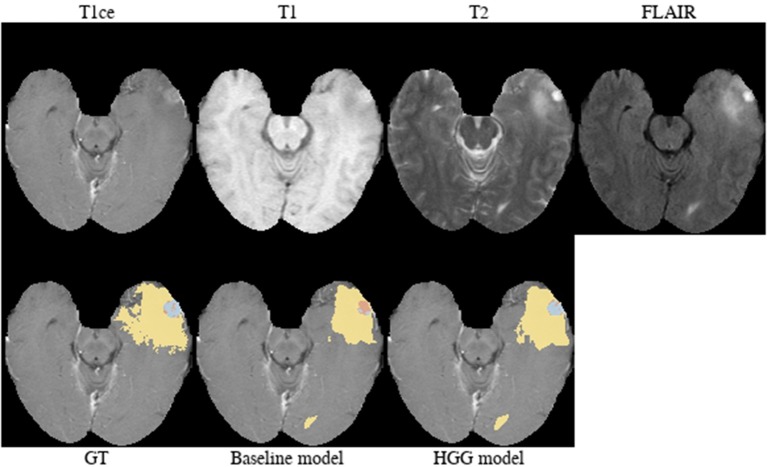
Brats18_TCIA01_425_1.

**Brats18_TCIA01_425_1** (Figure 9). The baseline model underestimated the subtle contrast-enhancement of this HGG case. We can speculate that in the situation of subtle enhancements the baseline model was biased more toward segmenting a tumor core with small enhancing foci, whereas the HGG model was capable of delineating the full extent of the contrast-enhancement.

**Figure 10:**
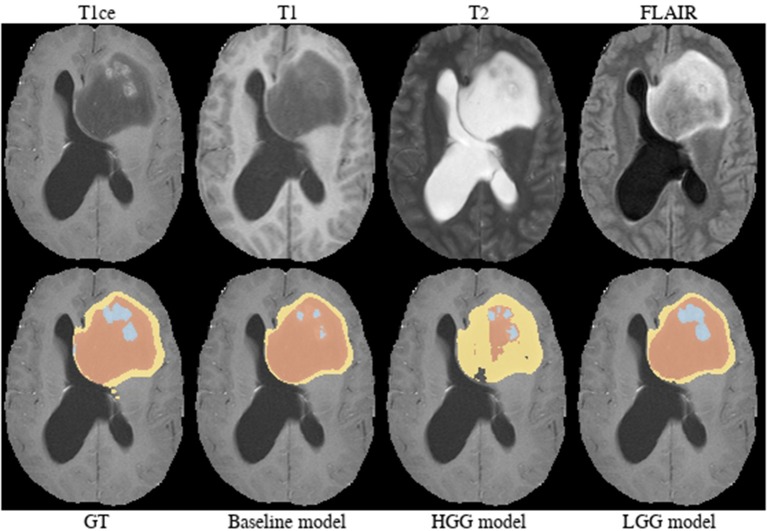
Brats18_TCIA05_444_1.

**Brats18_TCIA05_444_1** (Figure 10). The baseline model provided a better segmentation than the HGG model for this case. The tumor was indicated to be an HGG. The location of the tumor in the frontal lobe and its appearance exhibiting focal contrast enhancements and a large non-enhancing tumor mass are suspicious of a potential IDH-mutant glioblastoma. This would imply that it initially emerged from an LGG (called secondary glioblastoma). Applying the LGG model to the case significantly outperforms the HGG model (Figure 10), which would support the hypothesis of a mutated LGG.

While the previous analysis of cases was to some extent speculation, we can nevertheless condense three main, factual observations from it: First, individual segmentation results are strongly affected by the composition of the segmentation model's training data. Second, depending on the underlying factors that caused a given image appearance and segmentation ground truth, a given subset of the training data can actually improve the segmentation result compared to a baseline trained on all data. Third, disagreement (or joint failure) among segmentation models trained on different subsets of training data (Figure 3) may actually help in the identification of these underlying factors. Among the manually reviewed 18 cases with a large deviation between the two models, we observed issues with the input images (4 cases), potentially arguable ground truth (7 cases), and special imaging phenotypes (5 cases). Arguable ground truth is often attributed to edema that could be labeled as tumor core instead. Edema typically propagates along white matter and spares cortical ribbons as well as deep gray matter structures (Pope et al., 2005), while non-enhancing tumor leads to a distortion of the gray/white matter junction [cf. BRATS18_CBICA_BHB_1 (Figure 7) FLAIR with the case presented in Figure 3 of Lasocki and Gaillard, 2019].

## 4. Discussion

The title of the manuscript contains the phrase “Divide and Conquer,” where “Divide” refers to the stratification of training data. Data stratification and subsequent model training was employed as a simple, straightforward technique to include prior knowledge. We have proposed two ways of how to use data stratification to “conquer” brain tumor segmentation: First, the targeted application of a specialized model (HGG model) to the respective data (HGG test case). Second, the utilization of disagreement among specialized models' outputs and ground truth segmentations to identify outliers and possible latent factors hampering generalization.

Implicitly adding prior information to the models by stratifying the data by tumor type (HGG and LGG) seems to be beneficial for the segmentation of the tumor core for high-grade glioma. Yet, the LGG-only model, which was trained with fewer samples (*N* = 75) compared to the baseline model (*N* = 285), showed no statistically significant deterioration of the segmentation performance. A statistically significant improvement in 64.9% of the subjects for the tumor core is accompanied by a non-significant improvement of 54.6% for contrast enhancing and non-significant decrease (only 48.8% better-ranked subjects) for the whole tumor. It has been shown in multiple studies (Asari et al., 1994; Wiestler et al., 2016; Hsieh et al., 2017) that HGG and LGG tend to exhibit different qualitative and quantitative imaging features in structural MRI, involving heterogeneity of contrast enhancement, cystic components, intratumoral hemorrhage, and necrosis, which in context of tumor segmentation affect the definition of the tumor core greatly. Therefore, the stratification of the training data into HGG and LGG yields subsets with more homogeneous and consistent definitions of the tumor core. However, we presented also exceptions [e.g., BRATS18_2013_21_1 (Figure 4) in section 3.2] which actually profit from training data of opposite tumor grade.

In addition to improving segmentation performance, deep learning models trained on stratified data can be used to drive exploration of the training data. In section 3.2 we demonstrated that the disagreement between such models in relation to the ground truth data can assist in the identification of latent factors (e.g., imaging phenotypes) which may pose significant challenges in a deep learning model's capability to generalize across the complete problem domain. We argue that especially in a pathology as complex as brain cancer, the identification of such latent factors and their proper treatment in a deep learning model is of utmost importance to guarantee robust segmentation performance that satisfies clinical needs. In section 4.1 we provide propositions on how latent factors such as the tumor type could be treated in deep learning segmentation models beyond simple data stratification.

Our results demonstrated the potential of summary statistics (e.g., mean or median) to obfuscate significant differences between distributions of segmentation performance measures (e.g., Dice coefficient). These significant differences can be revealed through the calculation of a case-based ranking. Furthermore, case-based ranking enables the straightforward application of nonparametric statistics to detect significant differences with the advantage of more limited assumptions regarding the distribution of the data when compared to parametric statistics, and robustness to outliers. Case-based ranking also follows the narrative of precision medicine in which the identification of subpopulations of patients, who benefit from a medical intervention, based on experimental observations is central. It enables a more fine-grained analysis on the level of the patient and potentially an identification of patient subpopulations relevant for the task at hand.

Previously, Pereira et al. (2016) trained on data stratified into HGG and LGG. They employed two different Convolutional Neural Network architectures for patch-wise segmentation of HGG and LGG. In contrast, we hypothesized and demonstrated that a mere stratification of the training data into HGG and LGG without any changes to architectures or hyperparameters can lead to improved segmentation performance. Furthermore, their focus was on an ablation study of methodological components with respect to their two grade-specific architectures and their results were based on the BraTS 2013 Leaderboard dataset (21 HGG, 4 LGG cases) and BraTS 2013 Challenge dataset (10 HGG cases).

### 4.1. Outlook

With the rise of precision medicine and tailored therapies, the consideration of patient-specific information (e.g., genetics) becomes ubiquitous (Giardino et al., 2017). Leveraging data from multiple sources remains a challenge for the next generation imaging technologies (Kim et al., 2016), potentially requiring to rethink the *one size fits all* concept. For automatic brain tumor segmentation, various architectural and conceptual changes are imaginable beyond simple data stratification strategies.

By completely separating the data, each of the individual models has fewer data available for training, although with the benefit of a less heterogeneous domain (only one tumor type). Instead of implicitly adding the prior information of the tumor to the data by stratification, an alternative approach could be to explicitly add this information as input to the network. Particularly the first layers of the network might be less susceptible to the tumor type as filters for representation learning could share commonalities between both domains. By adding the information directly to the input layer or injecting it into the latent feature space might allow the network to intrinsically adapt the segmentation output according to the given tumor type.

A different approach would be to regard the problem of segmenting high-grade and low-grade glioma as a multiple-source adaptation problem. In this setting, the goal is to effectively combine base learners trained on multiple source domains in order to perform a prediction on a target domain, which can be any mixture of the source domains. In our case, the source domains would be subclasses of gliomas: either high-grade and low-grade data, or potentially a more fine-grained subdivision (e.g., WHO grade or classification). The target domain constitutes of a mix of different glioma cases. Recently, a number of theoretic and algorithmic contributions were made in the area of multiple-source adaptation (Hoffman et al., 2018; Zhao et al., 2018), which could be applied in the scenario of learning from multiple disease entities such as brain tumor types or grades.

The clinical importance of brain tumor segmentation for quantitative image analysis will only grow in the near future. Recently, various segmentation methods have been proposed which are capable of accurately delineating brain tumor compartments longitudinally (Weizman et al., 2014; Meier et al., 2016), perform assessment of treatment response (Huber et al., 2017; Kickingereder et al., 2019), are used for the purpose of radiomic analysis (Bakas et al., 2017c), and for performing planning of radiation therapy (Sharp et al., 2014; Herrmann et al., 2018; Agn et al., 2019; Lipkova et al., 2019). It is, therefore, necessary to provide automatic segmentation methods which are capable of robustly generalizing across different types or grades of brain tumors. Our methodology of training deep learning models on stratified training data is a straightforward approach to potentially improve the segmentation performance of already existing learning-based methods with regards to different tumor types.

In the light of our results and the trend toward precision medicine, we encourage challenge organizers to make information on the tumor type or grade available as additional input data, allowing teams to incorporate such prior information into their models.

### 4.2. Limitations

The evaluation is based solely on the BraTS training dataset (using cross-validation). Results for the official validation set are unknown since the required tumor type is not available for these data. Indeed we acknowledge that the tumor grade is usually not yet available on the first admission. However, we think automatic segmentation models will probably be employed first for retrospective studies, to assess the extent of resection in patients undergoing surgery (Meier et al., 2017), or to assess tumor progression postoperatively (Kickingereder et al., 2019) where tumor grades are usually known. First attempts have been made to classify tumor grades from MRI (Decuyper and Van Holen, 2019), which would allow identification of the correct model from imaging only. Alternatively, one might run such a segmentation algorithm twice: first for a rough identification of the tumor compartments and based on the result (e.g., presence of CE, ratio of compartment volumes, or manual review of the intermediate results by an expert) apply the specific model to get a refined segmentation.

The benefit of stratifying the training data has been shown with the model ranked third in the BraTS 2018 challenge (McKinley et al., 2019a). This particular model was chosen, as it was a top-ranked method in the most recent BraTS challenge (2018) that achieved its results using only a standard GPU and data from the BRATS challenge. The method ranked first (Myronenko, 2019) depended on a GPU with 32 GB of memory (to which most research groups do not have access), while the second-ranked method (Isensee et al., 2018) was co-trained with additional data (not including information about tumor grades). To what extent the proposed approach generalizes to other architectures remains an open question. Other models might suffer more from the reduction of training samples due to the stratification. The proposed architecture is known to be robust to fewer training samples (McKinley et al., 2019b).

## 5. Conclusion

Implicitly adding prior knowledge by dividing data into distinct domains can improve the performance of deep learning-based segmentation methods and compensate for the smaller number of samples available for training a model. The tumor grade has shown to be an important latent factor in the segmentation of gliomas. Comparing the performance of models by case-based ranking statistics may reveal significant differences that are otherwise concealed in summary statistics such as the mean Dice coefficient.

## Data Availability Statement

Publicly available datasets were analyzed in this study. This data can be found here: http://braintumorsegmentation.org.

## Ethics Statement

The study is based on a publicly available dataset from the Multimodal Brain Tumor Segmentation Challenge 2018 (BraTS, http://braintumorsegmentation.org/).

## Author Contributions

MReb, RM, RMc, RW, and MRey: design of experiments. MReb: perform experiments, data analysis. UK and RM: analysis of selected cases. MReb, RM, and RMc: results interpretation. MReb and RM: manuscript drafting, RMc: manuscript revision. All authors reviewed and approved the final version of the manuscript.

### Conflict of Interest

The authors declare that the research was conducted in the absence of any commercial or financial relationships that could be construed as a potential conflict of interest.
