# Supplementary material for: Divide and Conquer: Stratifying Training Data by Tumor Grade Improves Deep Learning-Based Brain Tumor Segmentation
Source: Front Neurosci. 2019 Nov 5;13:1182. doi: 10.3389/fnins.2019.01182 (PMC6848279; doi:10.3389/fnins.2019.01182)
Supplement: Supplementary file 1 [file Data_Sheet_1.PDF]

Supplementary Material

1 SUPPLEMENTARY FIGURES

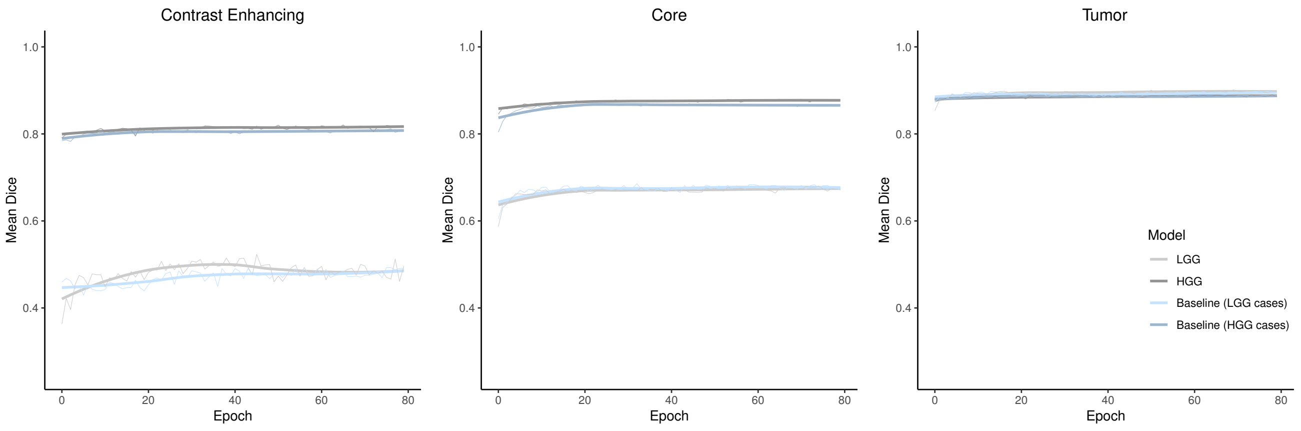

Figure S1. Progress of training for the three models. Average Dice of subjects in the validation set over all five folds from the cross-validation.

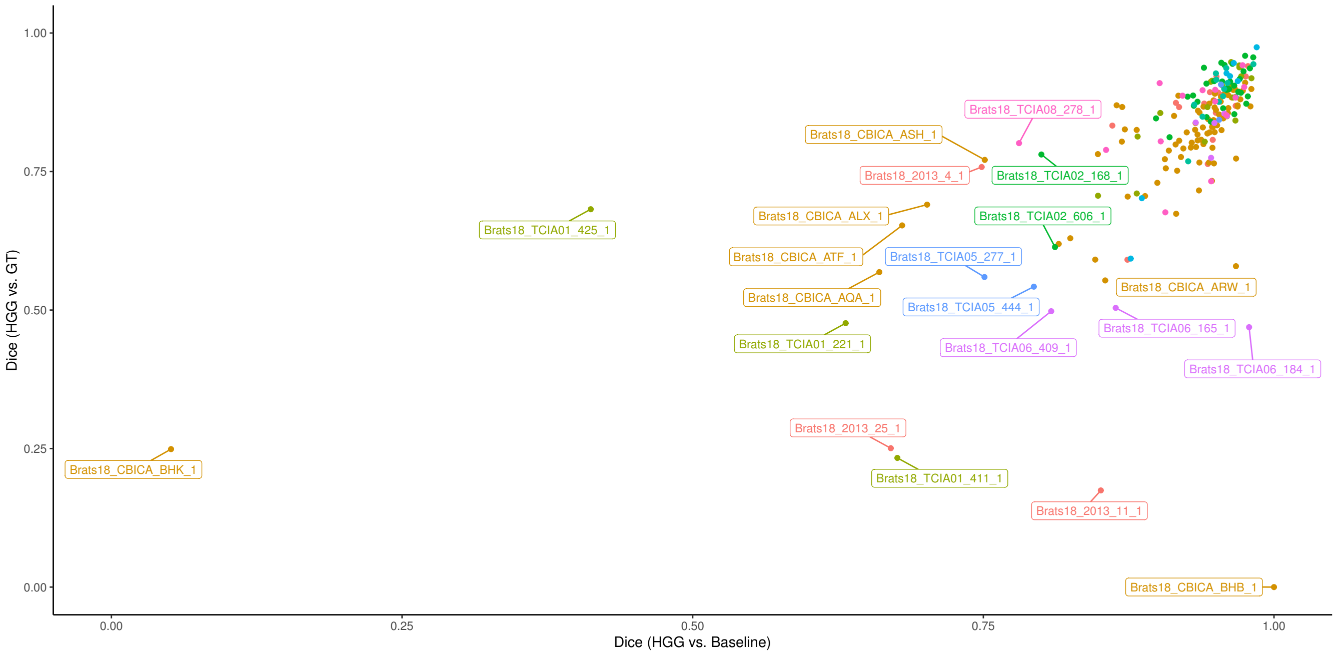

Figure S2. Performance of the HGG-only model for the **contrast enhancing** compartment (y-axis) and agreement with the baseline model (x-axis). Colors indicate the center (2013, CBICA, TCIA01-08).

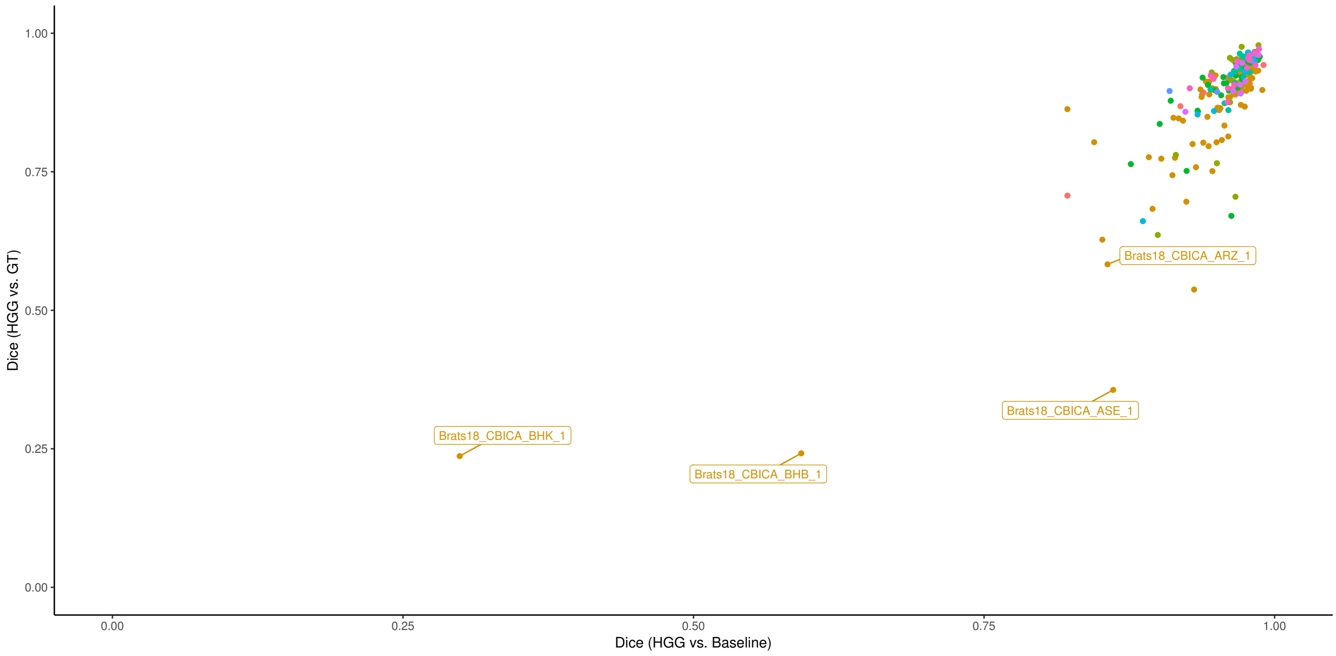

Figure S3. Performance of the HGG-only model for the **whole tumor** compartment (y-axis) and agreement with the baseline model (x-axis). Colors indicate the center (2013, CBICA, TCIA01-08).

2 SUPPLEMENTARY RESULTS

This section shows the segmentation for the remaining selected cases along with a qualitative analysis.

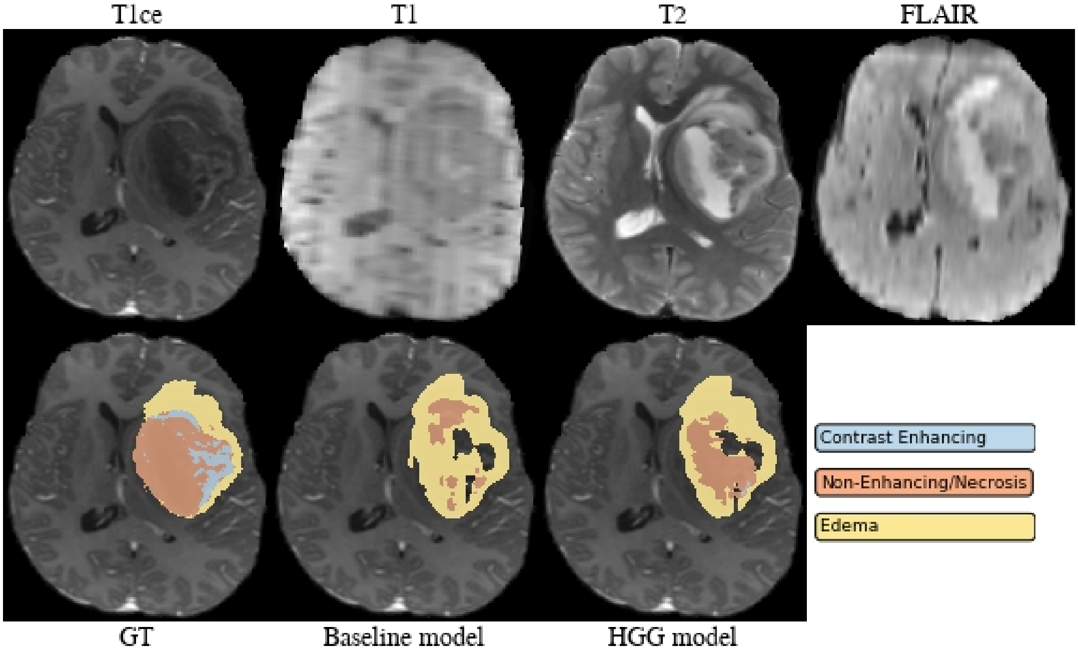

Figure S4. Brats18_2013_11_1: Both models performed badly for this HGG case, with the HGG-model capturing more of the tumor core. In particular, both models failed to identify the contrast enhancing part which might be due to the bad quality of the blurry T1 input image and the subtle enhancement. Contrast enhancing is usually defined by hyperintensities in the T1ce image compared to the native T1 serving as the reference.

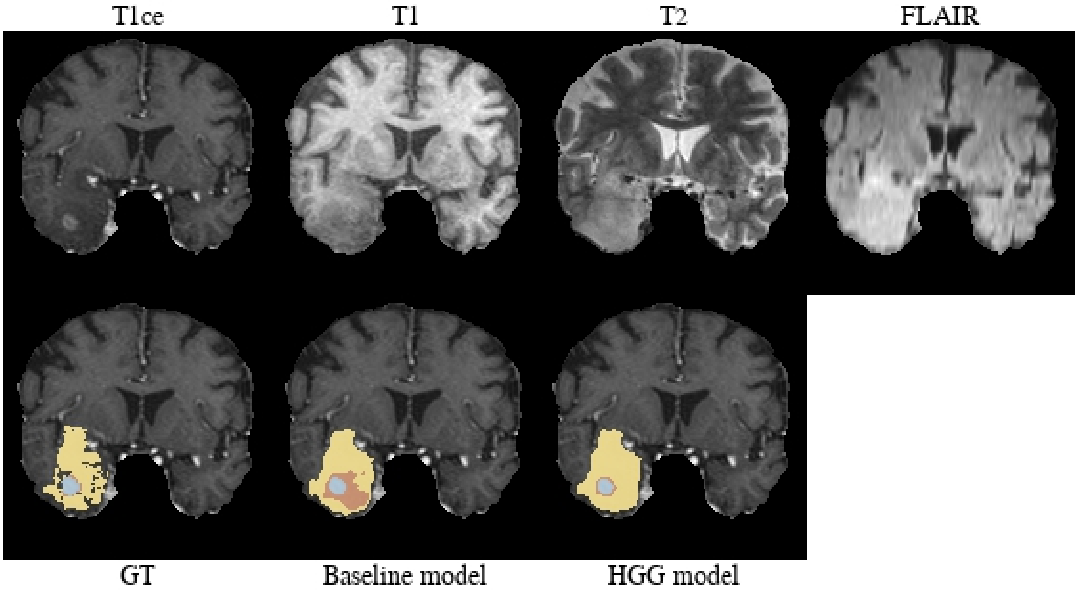

Figure S5. Brats18_CBICA_ABN_1: For this case, the HGG model achieved a better segmentation of the tumor core. However, we note that the edema demarcated in the ground truth is likely non-enhancing tumor. We base this observation on the same argumentation as for the cases Brats18_CBICA_AXJ_1 and Brats18_CBICA_BHB_1: The cortical space-occupying effect and the junction of gray/white matter which is strongly distorted.

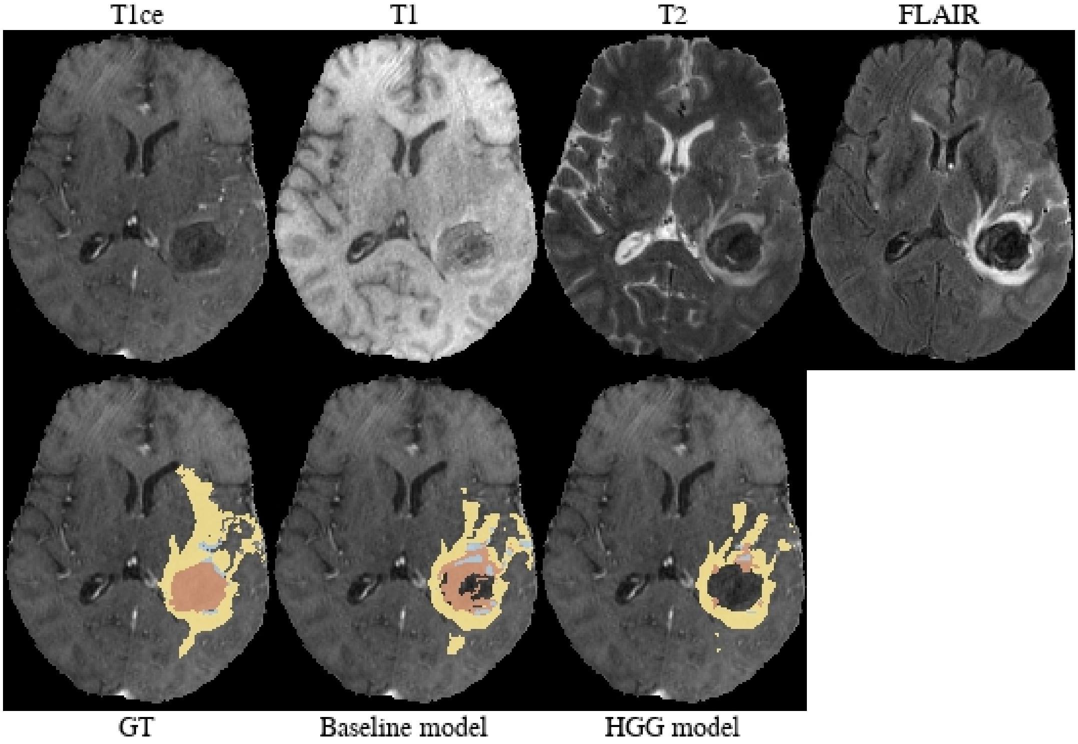

Figure S6. Brats18_CBICA_ATF_1: The baseline model achieved a superior segmentation result compared to the HGG model's output. This case is similar to Brats18_2013_25_1 in the sense that we can also observe a strong hypointensity in the T2-weighted images which might be suspicious of an intratumoral hemorrhage. Similarly, the baseline model was less affected by this and provided a more robust segmentation of the tumor core. In general, tumors exhibiting intratumoral hemorrhage are likely underrepresented in the BraTS dataset.

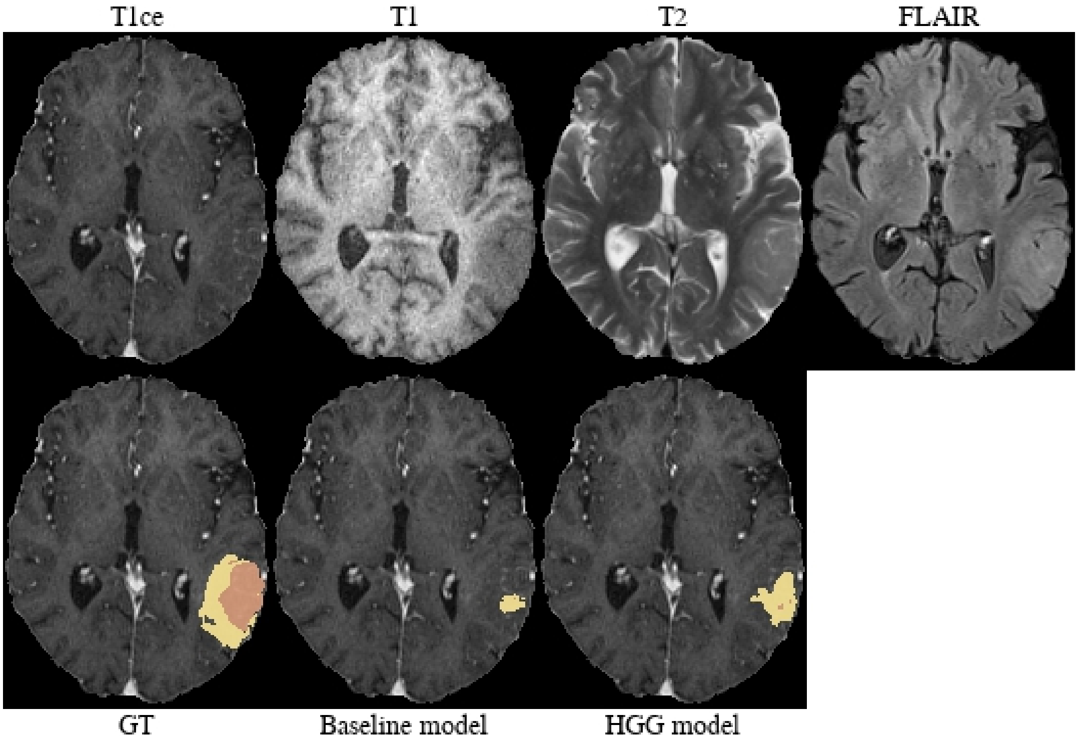

Figure S7. Brats18_CBICA_BHK_1: Both models failed to segment this tumor. In its visual appearance the tumor is similar to the one in Brats18_CBICA_BHB_1 exhibiting a rather diffuse T2-hyperintensity with a cortical space-occupying effect and distortion of the gray/white matter junction. For Brats18_CBICA_BHB_1 both models were not capable of segmenting the tumor as well. The segmentation of the edema in the ground truth seems to be rather arbitrary and we think that the complete lesion might in fact be non-enhancing tumor.

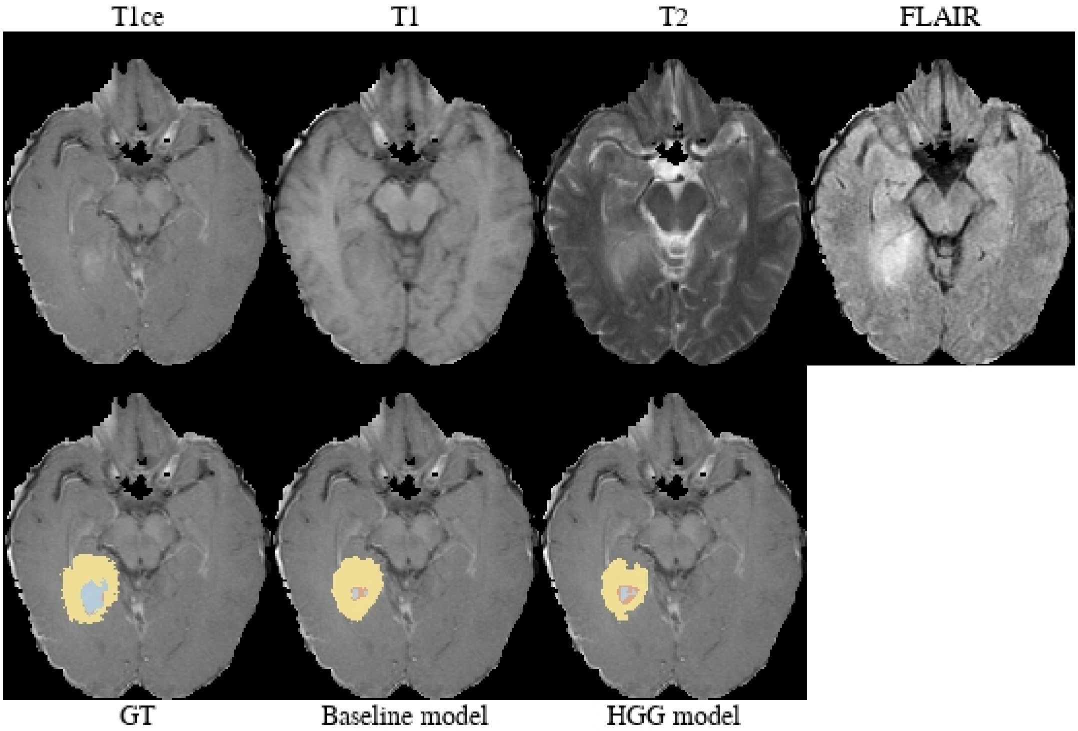

Figure S8. Brats18_TCIA01_411_1: Both models performed rather poorly with the HGG model presenting a slightly better overall performance. In particular, both models underestimated the subtle contrast-enhancement present in the image. It seems that the original imaging sequences had a large slice thickness and the interpolation to a standard 1 millimeter isotropic resolution further degraded the imaging quality. We think the poor segmentation performance of both models might be the result of the subtle enhancement which is further amplified by the degraded image quality.

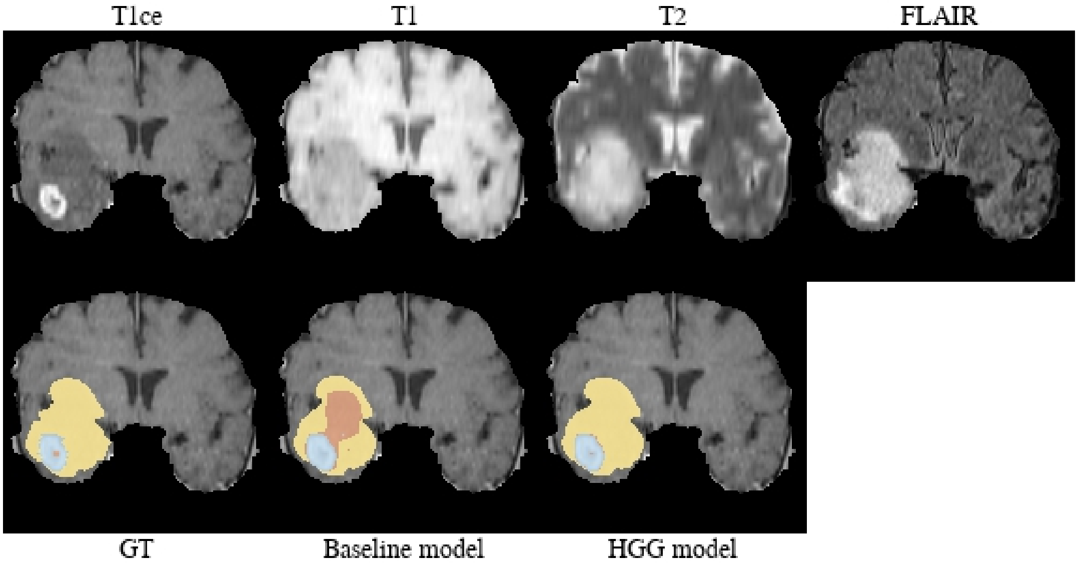

Figure S9. Brats18_TCIA02_171_1: Here the HGG model provided a segmentation result which is more consistent with the ground truth compared to the baseline model. The basline model overestimated the presence of non-enhancing tumor, which might be an effect of the additional low-grade glioma training samples. However, when looking at the ground truth segmentation, one can also argue that the edema has been overestimated and in fact large parts are non-enhancing tumor.

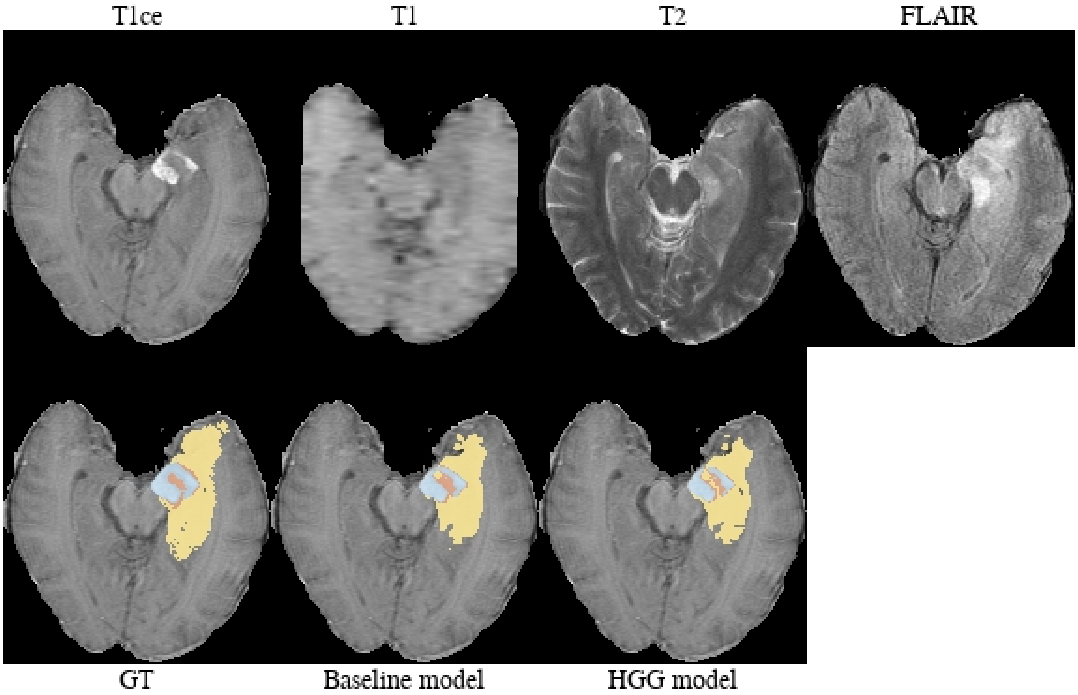

Figure S10. Brats18_TCIA04_343_1: For this case, the baseline model performed superior compared to the HGG model. From our point of view, large parts of the edema in the ground truth are in fact non-enhancing tumor. Additionally, the quality of the input images is rather bad.

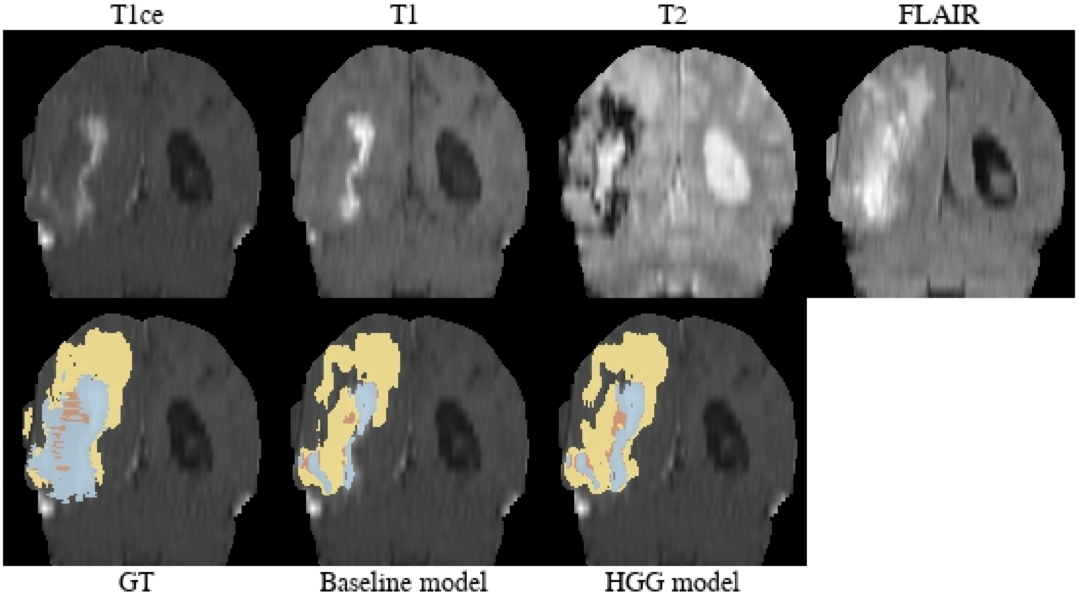

Figure S11. Brats18_TCIA05_277_1: Here the HGG model provided a more accurate segmentation for all three tumor regions. We can observe a composition of two bleedings, which originated at different time points in the past. The older bleeding is hyperintense in both the native T1-weighted sequence and the gadolinium-enhanced sequence and hypointense in the T2-weighted sequence. The younger bleeding is isointense in both T1-weighted sequences and hyperintense in the T2-weighted sequence. We can speculate that tumors exhibiting such a pattern are underrepresented in the BraTS dataset. The staircase-like effects in the ground truth indicate a manual segmentation exclusively in the axial direction.

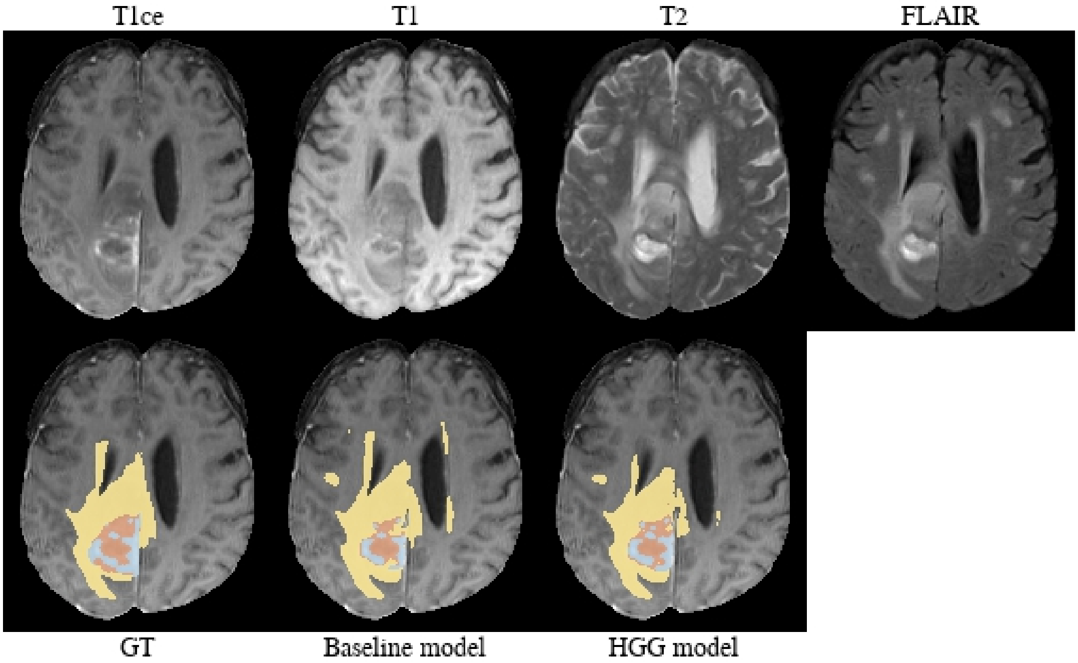

Figure S12. Brats18_TCIA06_409_1: Both models performed comparably for this case with a rather subtle enhancement.

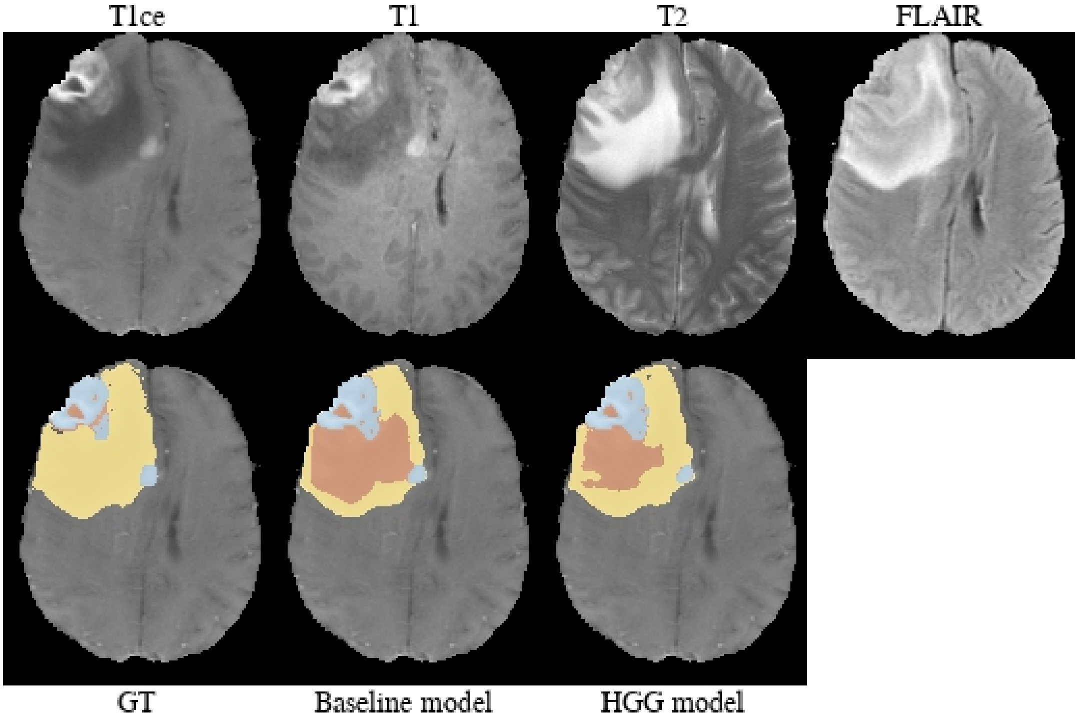

Figure S13. Brats18_TCIA08_113_1: The HGG model provided a better delineation of the tumor core for this example. It seemed to be less affected by the inhomogeneity of the FLAIR signal in the edema. Furthermore, the native T1-weighted image is in fact a T1-weighted image with contrast-agent.

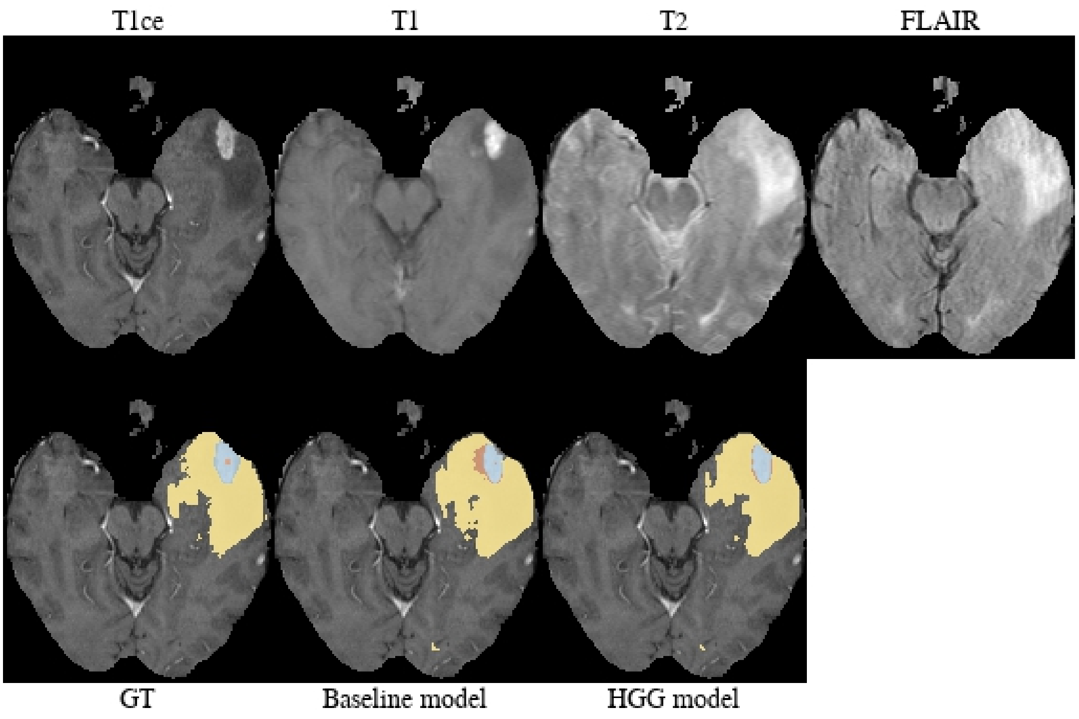

Figure S14. Brats18_TCIA08_406_1: For this particular case, the native T1-weighted image is in fact a contrast-enhanced T1-weighted image. Both models showed remarkably good performance given the fact they were fed with a wrong input sequence.
